# Effect of Operating Parameters on the Performance of Integrated Fixed-Film Activated Sludge for Wastewater Treatment

**DOI:** 10.3390/membranes13080704

**Published:** 2023-07-28

**Authors:** Sharjeel Waqas, Noorfidza Yub Harun, Nonni Soraya Sambudi, Kunmi Joshua Abioye, Muhammad Hamad Zeeshan, Abulhassan Ali, Aymn Abdulrahman, Loai Alkhattabi, Ahmad S. Alsaadi

**Affiliations:** 1 Chemical Engineering Department, Universiti Teknologi PETRONAS, Bandar Seri Iskandar 32610, Malaysia; kunmiabioye@gmail.com (K.J.A.); muhammad_22009523@utp.edu.my (M.H.Z.); 2 Department of Chemical Engineering, Universitas Pertamina, Simprug, Jakarta Selatan 12220, Indonesia; nonni.ss@universitaspertamina.ac.id; 3 Department of Chemical Engineering, University of Jeddah, Jeddah 23890, Saudi Arabia; aquddusi@uj.edu.sa (A.A.); aabdulrahman@uj.edu.sa (A.A.);; 4 Department of Civil and Environmental Engineering, College of Engineering, University of Jeddah, Jeddah 23890, Saudi Arabia; laalkhattabi@uj.edu.sa

**Keywords:** integrated fixed-film activated sludge, suspended growth process, attached growth process, membrane bioreactor, wastewater treatment

## Abstract

Integrated fixed-film activated sludge (IFAS) is a hybrid wastewater treatment process that combines suspended and attached growth. The current review provides an overview of the effect of operating parameters on the performance of IFAS and their implications for wastewater treatment. The operating parameters examined include hydraulic retention time (HRT), solids retention time (SRT), dissolved oxygen (DO) levels, temperature, nutrient loading rates, and aeration. Proper control and optimization of these parameters significantly enhance the treatment efficiency and pollutant removal. Longer HRT and appropriate SRT contribute to improved organic matter and nutrient removal. DO levels promote the growth of aerobic microorganisms, leading to enhanced organic matter degradation. Temperature influences microbial activity and enzymatic reactions, impacting treatment efficiency. Nutrient loading rates must be carefully managed to avoid system overload or inhibition. Effective aeration ensures uniform distribution of wastewater and biofilm carriers, optimizing contact between microorganisms and pollutants. IFAS has been used in water reuse applications, providing a sustainable and reliable water source for non-potable uses. Overall, IFAS has proven to be an effective and efficient treatment process that can provide high-quality effluent suitable for discharge or reuse. Understanding the effects of these operating parameters helps to optimize the design and operation for efficient wastewater treatment. Further research is needed to explore the interactions between different parameters, evaluate their impact under varying wastewater characteristics, and develop advanced control strategies for improved performance and sustainability.

## 1. Introduction

Domestic and industrial wastewater treatment processes ought to follow standards that help to ensure environmental sustainability [1,2]. Discharge quality, energy efficiency, process cost, nitrogen removal, phosphorous removal, biogas production, and nitrification/denitrification are the key parameters for wastewater treatment. The development of wastewater technology is critical to improving effluent quality, meeting environmental standards, and developing long-term sustainability [3]. As more restrictive legislation is imposed concerning wastewater treatment, it is inevitable that treatment processes will need to be redesigned to be more efficient, economical, and comply with the limits [4,5]. Consequently, an advanced biological process that guarantees complete treatment efficiency is required to achieve the demanded water quality [6,7,8].

Biological treatment consists of attached and suspended growth. Microbes are affixed to a solid surface for the former, while for the latter, the microorganisms are in suspension [9]. Trickling filters and rotating biological contactors (RBC) are typically attached growth processes, while the activated sludge process (ASP) and membrane bioreactor (MBR) are the common types of suspended growth processes [10]. Microorganisms adhere to a medium in the attached growth process, developing a biofilm. As the wastewater comes in to contact with the biofilm, the microbes ingest the biodegradable organic matter to produce sludge, water, carbon dioxide, and other chemicals [11,12]. A secondary clarifier is typically connected at the end of the biological reactor to settle suspended sludge [13].

The integrated fixed-film activated sludge (IFAS) is a hybrid suspended growth-fixed film system extensively utilized in domestic and industrial wastewater to meet stringent effluent quality standards [9,14,15]. The IFAS removes dissolved organics from the wastewater and ensures complete nitrification–denitrification [16]. The complete removal of nitrogen through nitrification–denitrification requires high aeration intensity, extensive pumping capacity, large basin volumes, and often supplementary carbon sources. Suspended growth is crucial in biological oxygen demand (BOD_5_) removal, while the biofilm is responsible for nitrogen removal [17,18].

The IFAS is a hybrid process that”emer’ed in 1994 as a leading-edge option that provides an ecological solution for treating municipal and industrial wastewater [19,20]. A moving bed biofilm reactor (MBBR) uses different types of media suspended in the bioreactor with the help of aeration. It uses return activated sludge (RAS) to grow the microorganisms in the suspended form as in the ASP [21,22]. The bio-carrier is kept inside with the help of the screens installed at the reactor’s discharge. By permitting the development of activated sludge by returning the RAS, the microorganisms are composed of biofilm and well-mixed flocs [23]. Inserting media in the bioreactor increases the treatment capacity by introducing suspended and attached growth processes. It increases the treatment capacity, loading rate, and effluent quality without requiring extra tank volume [24]. All existing ASPs can be upgraded to IFAS to improve the treatment capacity and plant retrofits. A higher loading rate, lower retention time, and smaller basin volume demonstrate superior effluent quality compared to ASPs [9]. The IFAS accomplished enhanced nitrogen removal thanks to nitrifying bacteria inside the carrier media. Full-scale enhanced nitrogen removal has been successfully achieved with it due to its compactness and robustness. Enhanced phosphorus removal has been accomplished for the pilot scale. The IFAS improves the effluent quality compared to an ASP and depicts greater stability, reduced solids loading and reduced sludge production, and enhanced sludge settleability [25].

This review aims to provide an overview of the effect of operating parameters on the performance of IFAS for wastewater treatment. Key operating parameters, including hydraulic retention time (HRT), solids retention time (SRT), dissolved oxygen (DO) concentration, temperature, pH, and nutrient loading rates, are discussed. The review highlights the importance of monitoring and controlling these operating parameters to achieve optimal performance in IFAS.

## 2. Basic Concept of IFAS Technology

**Figure 1:**
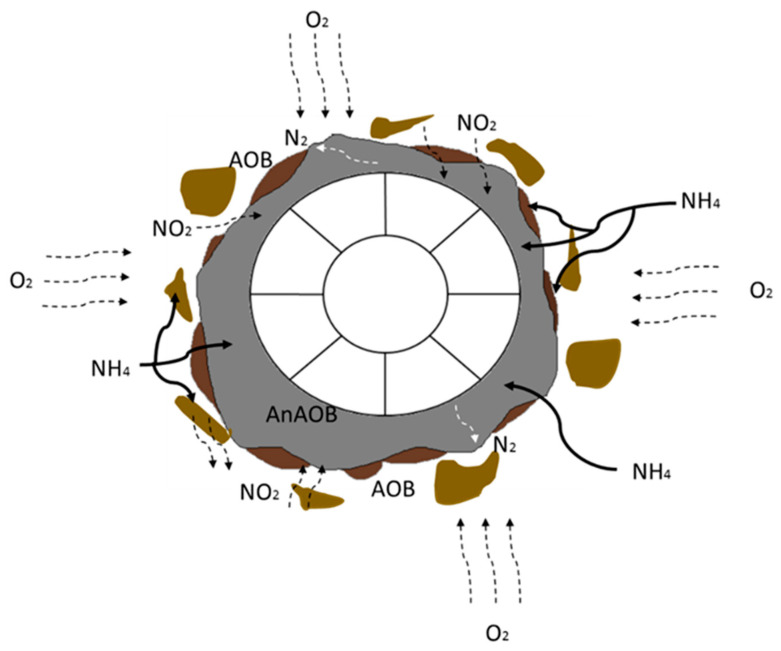
Basic mechanism of IFAS showing the microbial activity.

IFAS combines suspended growth-activated sludge with a biofilm process. The basic concept of the IFAS technology is to increase the surface area available for the growth of microorganisms involved in the treatment process, thereby increasing the treatment capacity without increasing the reactor size. The basic mechanism of microbial activity for the degradation of organics and nutrients is shown in Figure 1.

**Figure 2:**
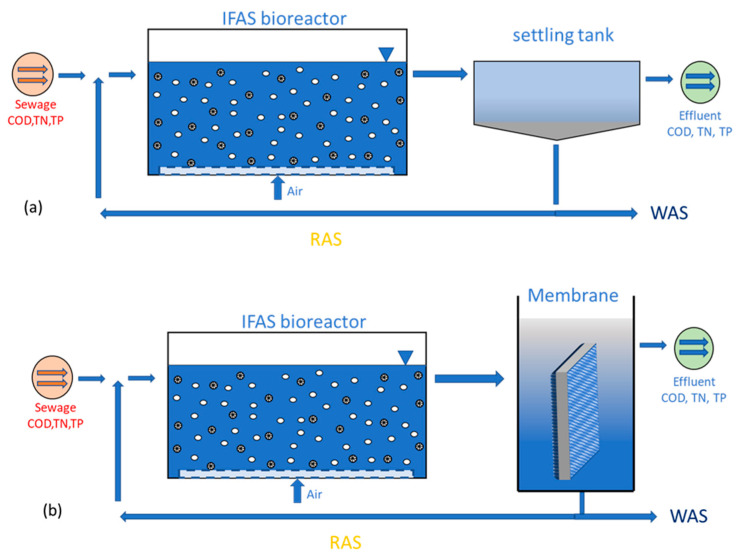
Schematic diagram of (**a**) IFAS with settling tank and (**b**) IFAS with membrane technology.

Figure 2 shows the schematic diagram of two different configurations of IFAS. IFAS supported with a settling tank combines a biofilm carrier and suspended floc followed by a settling tank. In IFAS, the wastewater flows through a reactor containing suspended and attached growth media. IFAS combines suspended and attached growth bacteria, offering better chemical oxygen demand (COD) removal and complete nitrification [9,26]. The suspended growth media consists of suspended microorganisms that consume organic matter and nutrients in the wastewater. The attached growth media, on the other hand, consists of a fixed media surface that supports the growth of microorganisms in a biofilm. The attached growth media is typically made of plastic, ceramic, or other materials with a high surface area. The media is designed to have a high void fraction, allowing for microorganisms’ growth and good oxygen transfer. The biofilm attached to the media surface provides additional surface area for the growth of microorganisms that oxidize or break down contaminants in the wastewater.

### 2.1. Advantages of IFAS

The IFAS offers several advantages in wastewater treatment. The IFAS allows for the growth of a biofilm on the fixed media, which increases the effective surface area available for microbial activity. The increased surface area allows for a higher biomass and enhances the system’s treatment capacity without requiring a larger reactor volume. The biofilm in the IFAS promotes the growth of nitrifying bacteria, which are responsible for converting ammonia to nitrate through nitrification. It effectively removes nitrogen from the wastewater, addressing concerns related to eutrophication in receiving water bodies [27]. The presence of the biofilm in IFAS provides a stable environment for microorganisms. The biofilm acts as a protective barrier, offering resilience against fluctuations in influent characteristics and shock loads, resulting in improved process stability and robustness [11].

The IFAS is easily retrofitted into existing ASPs, making it a cost-effective option for upgrading conventional wastewater treatment. The addition of a carrier media integrates into the existing treatment process without significant modification. The IFAS achieves high treatment efficiency in a relatively compact footprint. The increased biomass in the biofilm allows for a more efficient use of reactor volume, resulting in reduced space requirements compared to traditional ASPs. The combination of suspended growth flocs and an attached growth biofilm enhances the settling characteristics of the sludge. The formation of larger, denser flocs improves settling rates, resulting in better solid separation and reduced sludge production [28,29].

The biofilm in IFAS offers protection to microorganisms against toxic substances. The attachment of microorganisms to the carrier media provides a buffer against the adverse effects of toxic compounds, allowing for improved treatment performance. It operates with lower energy requirements than other advanced treatment technologies [30]. The increased biomass concentration and enhanced treatment efficiency contribute to reduced energy consumption for aeration and mixing processes. It combines the benefits of suspended and attached growth processes, resulting in improved treatment performance, increased treatment capacity, and enhanced process stability. These advantages make IFAS a favorable option for various wastewater treatment applications.

### 2.2. Disadvantages of IFAS

The IFAS offers several advantages and has a few disadvantages that should be considered. The carrier media in IFAS becomes clogged with biomass and other particulate matter over time. This reduces the effective surface area for biofilm growth and limits the treatment capacity. Regular maintenance and cleaning of the media are required to prevent clogging and maintain optimal performance. Depending on the type of carrier media used, there is a possibility of media degradation over time. Factors such as exposure to chemicals, high temperatures, or abrasive conditions cause wear and deterioration of the media. Media degradation impacts stability and performance and may require media replacement [31].

The IFAS generally requires a more complex design and operation compared to conventional ASPs. Adding carrier media and associated equipment, such as media retention screens or settlers, adds complexity to the system. Proper design, monitoring, and maintenance are crucial to ensure optimal performance, which may require additional expertise and resources—incorporating carrier media and associated equipment results in higher capital and operational costs. The carrier media cost, media retention devices, and additional monitoring equipment should be considered during planning and implementation [32].

In certain operating conditions, such as high hydraulic shear forces or excessive air scouring, there is a risk of biofilm detachment from the carrier media. Dislodged biofilms negatively impact downstream processes, reduce treatment efficiency, and increase suspended solids. Nitrification, converting ammonia to nitrate, is generally slower at lower temperatures. IFAS may experience reduced nitrification efficiency during colder periods, which impacts the overall nitrogen removal performance [33].

While IFAS effectively removes nitrogen and organic matter, it may have a limited phosphorus removal capacity. Other treatment processes or modifications may be necessary to achieve adequate phosphorus removal if required by the effluent discharge limits [34]. It is important to note that the specific disadvantages can vary depending on the design, operational practices, and particular conditions. Proper planning, design and regular monitoring help mitigate these disadvantages and ensure the successful implementation of IFAS technology.

## 3. Operating Parameters of IFAS

Recently, many researchers have investigated the performance using different parameters [17,35]. The performance based on removing nutrients and organic matter has shown promising results in treating domestic, municipal, and industrial wastewater. The IFAS was developed as a compact and novel treatment process and alternative to the ASP [6,36]. IFAS was incorporated by reducing the sludge production, having better settleability characteristics, and less fouling than MBR and MBBR [37,38]. The IFAS is designed with a settler or MBR to achieve the desired effluent quality. The addition of suspended biomass in the IFAS is compared to MBBR, which only uses carrier media [39].

The performance parameters are essential in degrading conventional pollutants [40,41,42]. The design parameters and operating conditions considerably affect efficiency. The design should consider the media type, placement, aeration pattern, filling ratio, operating parameters, and forward flow rate [43]. The bacterial growth is dependent on the hydrophobicity of the floc [44]. Faster-growing microorganisms have higher hydrophobicity. Studies conducted on pure culture growth have depicted that suspended flocs have higher hydrophobicity than biofilm growth [45,46]. The performance of IFAS is influenced by various factors that should be carefully considered during system design, operation, and maintenance. Understanding these factors is essential to optimize performance and achieve treatment goals.

### 3.1. Carrier Media Filling Ratio

IFAS utilizes carrier media for biofilm development to treat wastewater. The carrier media must be carefully designed to provide a high internal surface area and density lighter than water to float through aeration easily [47]. Various materials are used for the biofilm-attached growth of bacteria. Specific surface area (SSA) measured as m^2^/m^3^ is the primary parameter for determining total performance. The carrier media is important as it contributes to particular characteristics of microbial growth that help in organics removal, nitrification/denitrification, and phosphorous removal from domestic, municipal, and industrial wastewaters [48,49].

The filling ratio is an important parameter for both MBBR and IFAS, which plays an important role in controlling the microbial activity in the bioreactor. It has been observed that the filling ratios range from 10% to 60% depending on the influent wastewater concentrations and the presence of various compounds [9]. The hydraulic retention time (HRT) varies from very few hours to several days, depending on the strength of the wastewater being treated to fulfill effluent standards for organic matter and nutrient removal [7]. The IFAS operates at a lower SRT compared to the MBBR due to the presence of both suspended and attached microorganisms. The addition of 20% carrier media to the bioreactor improves the organic removal efficiency [50]. The SRT decreases from 8 to 4 days, thus improving the settleability [51].

IFAS was employed to treat oil sands process-affected water (OSPW), and a filling ratio of 60% was applied using polyethylene (PE) carriers under various HRT of 48, 72, and 96 h. The effluent quality resulted in COD and an acid extractable fraction (AEF) removal of 56.83% and 51.51%, respectively, while treated in ozonated OSPW-IFAS [52]. A pilot-scale continuous plug flow and full-scale IFAS to treat high ammonium wastewater uses 15% and 20% polyester sponge (cubic) [53]. An IFAS-sequencing batch reactor (SBR) reactor with 20% carrier media and 8 h HRT was used to treat synthetic wastewater with silver nanoparticles. The system was run under COD 476.6 ± 20.2 mg/L, ammonium 29.5 ± 0.57 mg N/L, and total phosphorous (TP) 4.7 ± 0.2 mg/L. The IFAS-SBR achieved excellent removal efficiency for COD 96.8 ± 0.7%, ammonium 99.3 ± 0.3%, and TP 99.1 ± 1.3%. The treated effluent results indicate that the presence of nanoparticles does not cause any adverse effects on the removal of organic matter, ammonium, nitrogen, and phosphorous [54].

Two hybrid IFAS-MBR filled with PE carrier media and a filling ratio of 50% were employed in both systems with an HRT of 11.6–14.45 h and 10–20 days SRT to obtain >98% COD removal for both systems [55]. A simultaneous partial nitrification, anammox, and denitrification (SNAD)-IFAS for the ammonium-rich synthetic wastewater with non-woven polyester rings (density 1.1 g/cm^3^) was used as the carrier media with a 40% filling ratio and 0.75 d HRT and 20 d SRT employed for 120 days. The effect of pH on the microbial community was investigated, and it was concluded that pH shift affects small changes in microbial community structure [56]. The IFAS reactor operates under different dissolved oxygen (DO) levels to test the treatment performance and fluctuations in microbial community characteristics. Different DO concentrations (0.5, 2.5, and 4.5 mg/L) were applied, containing polypropylene carrier media running at 11.1 h HRT. The results confirmed the efficient COD removal up to 81, 90, and 94% for DO maintained at 0.5, 2.5, and 4.5 mg/L, respectively. The optimized value of DO was found to be 4.5, 4.5, 2.5, and 2.5 mg/L for organics, nitrification, denitrification, and total nitrogen (TN) removal, respectively. The suggested DO value was 4.5 mg/L for the high organic removal and reduced sludge production with a high SRT [57].

The lab-scale and full-scale plants were studied, filled with K5 plastic carrier media at 43% and 50% filling ratios. The HRT was set at 20–24 h and the SRT at 5 d (±2 d) for both systems. High ammonium concentration wastewater was fed to the system with inlet values as NH_4_^+^–N 907 ± 200 mg N/L, COD 414 ± 150 mg/L, BOD_5_ 29 ± 5 mg/L, and TSS 400 ± 200 mg/L for the lab-scale unit. Nitritation by AOB activity occurs in the suspended sludge (93% of the total AOB). The abundance of anaerobic ammonia-oxidizing bacteria (AnAOB); 96% of the total AnAOB, conducts the denitrification. The physical non-clogging sieve separating the AnAOB biofilm carrier and the AOB suspended sludge is a clear advantage to retaining AnAOB in the bioreactor [58].

The carrier media provides a longer SRT than ASPs to facilitate the evolution of nitrifying bacteria and stimulate nitrification [59]. Nitrifying bacteria responsible for the nitrification/denitrification depict different retention comportment. The nitrifying microorganisms preferred to stay in the biofilm rather than the sludge floc. The microorganisms attached as a biofilm provides longer SRT than the suspended flocs, and consequently, nitrifying bacteria grow slowly [59,60]. The phosphorous-accumulating organisms (PAO) are responsible for the degradation of phosphorous compounds as they tend to stay in the suspended floc where the aerobic, anoxic, and anaerobic conditions favor metabolism [28].

### 3.2. Hydraulic Retention Time

HRT is an important factor that determines the performance of the IFAS. Hydraulic loading is another important parameter directly determining HRT, influencing the reactor size [61,62]. It directly affects the performance efficiency by controlling the hydraulic loading. An optimized value of HRT needs to be determined for every system to operate at maximum removal efficiency [63,64]. It is depicted that low-strength influent wastewater normally requires a low HRT in the range of 3 to 12 h to obtain the desired effluent quality. High-strength wastewater and wastewater containing special compounds may require large HRTs to attain the mandatory sewage quality. However, HRT is purely based on the influent wastewater, operating parameters, discharge quality requirement, research objectives, and experimental design [65].

Heterotrophic species containing mixed ordinary heterotrophs and PAO show higher activity in the suspended sludge than biofilm carrier media [66]. Simple MBBR does not offer this behavior of growth of PAO in the suspended sludge. This is due to the specialization of microorganisms for both suspended growth and biofilm. The high removal rates show the suspended biomass has a greater affinity for organic matter. The biofilm on the carrier media develops nitrifying bacteria due to the longer SRT degrading ammonium to nitrite. Seeding occurs when the biomass is segregated from the carrier media and grows in the suspended sludge. This is only observed in IFAS, not the conventional ASPs or MBBRs. The growth of heterotrophs is much higher than that of autotrophs, confirming the higher growth rates and greater affinity to degrade the organics [67,68].

A lab-scale IFAS that was used to treat OSPW to reduce COD, ammonium, nitrate, and AEF was studied at a 30% carrier filling ratio, 48 h HRT and 43 d SRT in a 2 L bioreactor. The results indicated that COD, ammonium, nitrate, and AEF reduced from 317 ± 10.5 mg/L, 30.8 ± 0.9 mg/L, 0.94 ± 0.14 mg/L, and 88.62 mg/L to 177.7 ± 6.1 mg/L, 0 mg/L, 24.90 ± 1.58 mg/L, and 77.93 mg/L, respectively. The results indicate that the optimum performance of the bioreactor is obtained at 48 h HRT and 43 d SRT [69]. It concludes that IFAS is a promising solution for OSPW treatment. The free nitrous acid inhibition of biological phosphorous removal utilized Anox Kaldnes, K1 carrier media with a 12 h HRT and a 10 d SRT in a lab-scale 3 L batch reactor [70]. The results show that an optimum phosphorus removal is obtained at 12 h HRT and 10 d SRT. It is important to note that the optimal HRT may vary depending on the specific contaminants present in the wastewater, the desired effluent quality, and the available resources. Therefore, thoroughly understanding the wastewater characteristics and treatment goals is crucial for determining the appropriate HRT in IFAS. By selecting an optimal HRT, the IFAS achieves efficient pollutant removal and contributes to sustainable and effective wastewater treatment.

### 3.3. Biofilm and Flocs

Biofilm and flocs play important roles in the treatment process, each contributing to removing organic matter and other contaminants. Biofilms and flocs are distinctive microbial structures [40,71]. The biofilm refers to the layer of microorganisms that attach to the fixed media surface in the attached growth region of the reactor. The biofilm provides a high surface area for the growth of microorganisms and allows for removing a wide range of contaminants. The biofilm typically consists of diverse bacteria, fungi, and protozoa, which work together to break down and remove organic matter and nutrients [72]. Biofilm is highly effective at removing contaminants from wastewater because it provides a protective environment for microorganisms. The biofilm protects the microorganisms from predation by protozoa and other organisms and from changes in the wastewater quality. The biofilm allows for the growth of nitrifying bacteria, which play a critical role in removing nitrogen from the wastewater through nitrification [73].

The effect of different operational parameters on bacterial community growth must be understood to promote the effectiveness of IFAS [74]. The effluent nitrate concentration of 30 mg nitrate/L indicated the occurrence of nitrification. This effluent nitrate concentration was achieved when the C/N ratio was 10:1 [75]. The denitrification occurs under anoxic conditions when aeration is stopped for 40 min to facilitate sludge settling, where nitrogen gas is produced as the product. As the C/N ratio decreases, nitrogen gas production decreases due to less organic carbon being available for heterotrophic bacteria. The inside deeper part of the carrier media containing the biofilm and the inner layer of suspended sludge flocs might be anoxic to promote denitrification [76,77]. An interesting phenomenon occurred under the limiting C/N ratio. The nitrification rate of suspended sludge was much higher than in the conventional ASP, when both were working under the same operational parameters. This phenomenon is called the seeding effect and is attributed to biofilm detachment from the carrier media. The detached biofilm and heterotrophic bacteria attached to the carrier media are now suspended in the mixed liquor, resulting in increased heterotrophic bacteria in the suspended sludge. Various researchers have confirmed this seeding phenomenon, resulting in increased nitrification rates in the IFAS compared to ASPs [75,78,79].

In the suspended growth region of the IFAS, floc refers to a cluster of microorganisms that form in the wastewater as a result of the mixing and aeration [80]. Flocs are composed of a mixture of microorganisms, including bacteria, protozoa, and fungi, and are responsible for removing organic matter and nutrients. The flocs are highly effective at removing contaminants from the wastewater because they provide a large surface area for the attachment of microorganisms. The flocs also promote the formation of larger particles, which are easier to settle out. This settling process removes solids and other contaminants from the wastewater. The role of the biofilm and flocs in IFAS is critical to the system’s effectiveness in wastewater treatment. Biofilm provides a high surface area for the growth of microorganisms, while the flocs promote the formation of larger particles that can be easily removed from the wastewater. Together, these two components of the IFAS work in tandem to provide an efficient and effective means of treating wastewater.

### 3.4. Extracellular Polymeric Substance in Biofilm and Suspended Floc

EPSs promote the adhesion properties of the microbial cells to the solid surface by altering the surficial properties of the cellular surface of the cells [81]. The major components of an EPS are usually proteins and carbohydrates [82,83]. Other components, such as nucleic acids, lipids, inorganic components, and uronic acids, have been found in EPS in the different matrixes [83,84,85]. EPS production is required to adhere the microorganisms to the solid surface. Excessive production of EPSs could result in viscous bulking, hindering the organics’ diffusion and collapsing the complete degradation [86]. High biomass on the carrier media may result in higher EPS production [87]. The complex relationship between the EPSs and the C/N ratio needs to be studied to evaluate the system’s performance.

The settleability of sludge is determined by the polysaccharide/protein ratio that constitutes the EPS [88]. The composition of EPSs in the biofilm and floc has been studied by different researchers who found that the polysaccharide/protein ratio was greater in the biofilm than in the suspended activated sludge in the bioreactor [75,89]. The EPSs in the biofilm and floc possess different individualities due to different compositions, flocculation capacity, dewatering ability, and settleability [89]. The high polysaccharide/protein ratio for the EPS in biofilm is because the polysaccharides support microorganisms to attach to the solid surface [90]. The polysaccharides/protein ratio changes contrarily for biofilm and floc aggregates. The polysaccharides/protein ratio phase is attributed to high EPS contents and better sludge settleability. In the biofilm, the polysaccharides/protein ratio decreased from 17.1 ± 0.1 to 10.2 ± 4.6 first and then increased from 10.2 ± 4.6 to 20.7 ± 2.5 later [75]. Increasing the C/N ratio has no effect on the EPS content. Decreasing the C/N ratio from 20 to 4 caused an increase in EPS content from 5.6 to 9.3 mg total carbon content (TCC)/g of suspended solids (SS) for 5 days. A reverse trend is observed in the activated sludge of the bioreactor, where the polysaccharides/protein ratio decreases with the decrease in the C/N ratio [91].

The IFAS wastewater treatment plant at Hopedale, US, operates for ammonium removal at low temperatures of 7–9 °C. The DO was maintained at 4–6 mg/L to provide sufficient aeration for the biological activity and mixing. The effluent nitrate concentration was measured below the limit and remained within the limit even under low-temperature conditions. The attached biomass helps to maintain system performance due to the abundance of microorganisms. The effluent COD and TSS are averaged at 5 to 7 mg/L compared to the 15–20 mg/L values attributed to the previous ASP. The average SVI in the IFAS was 100 mL/g compared to the 150 mL/g in the ASP [92].

IFAS is operated to treat synthetic wastewater abandoned of aromatic amine compounds. The system was used at different HRTs (8, 24, 28, and 72 h) with varying influent COD concentrations (100 to 3500 mg/L). The results indicated that 90% removal was obtained for the COD influent concentration of 750 mg/L [93]. High ammonium wastewater treated in IFAS for pilot-scale and full-scale plants was observed in the anaerobic ammonium oxidation (anammox) biofilm and nitrifying activated sludge in the IFAS rapidly treating autotrophic nitrogen compounds [53]. The IFAS bioreactor cultivates granular sludge by biofilm detachment. The concentration in granular sludge was four times (>100 lm) higher than the anammox bacteria in flocculent sludge (<100 lm). Due to high DO concentrations and sufficient substrate levels, AOB responsible for converting ammonium to nitrate grow in the suspended sludge [94].

AOB profusion was substantially superior in the activated sludge samples than in the anammox bacteria. The AOB were 54.6%, while anammox bacteria were only 2.6%. The biofilm contains a significantly higher abundance of anammox than AOB. Anammox bacteria were present in a huge proportion (87.8%). In comparison, AOB were only 2.4% of the bacteria due to high DO concentrations and sufficient substrate levels in the activated sludge [9].

A small-scale IFAS reactor was operated to study the environmental performance and microbial investigation of treating municipal wastewater. The reactor was operated at HRT 6.9 h, SRT 7 d, DO~3 mg/L with biotexil cleartec carrier media made of polypropylene fabric. The system obtained average removal efficiency for COD~92%, BOD_5_ 91%, TSS 90%, TN 88%, and TP 50% while operated under optimized conditions. Microbial community analysis indicated that *E. coli* which constitutes >99% of all pathogens (*Salmonella* spp., *E. coli*, and *Shigella* spp.) was removed. Respirometric characterization of the activated sludge indicated that attached biomass dominates the overall removal of pollutants [72].

### 3.5. Carrier Media Selection

Carrier media selection is a crucial aspect of designing an IFAS for wastewater treatment. The choice of carrier media directly impacts the surface area available for biofilm growth, biomass retention, and overall treatment efficiency. Several factors should be considered when selecting the appropriate carrier media [95]. The carrier media should provide a large surface area to support the growth of biofilms and facilitate the attachment of microorganisms. The higher surface area allows for increased microbial activity and enhances the treatment capacity of the IFAS. Media with a greater surface area-to-volume ratio can accommodate a larger biomass and improve treatment efficiency. The carrier media should have sufficient porosity and void space to circulate wastewater and air within the system properly. Adequate void space ensures optimal oxygen transfer, nutrient diffusion, and hydraulic performance. Media with high porosity promote uniform distribution of wastewater and prevent clogging or channeling issues [28].

The carrier media should be durable, resistant to chemical and biological degradation, and have a long service life. This ensures that the media can withstand operational conditions, mechanical stress, and cleaning procedures without deteriorating or losing effectiveness. Long-lasting carrier media reduce maintenance requirements and ensure consistent treatment performance. The cost of the carrier media, including procurement, installation, and replacement, should be evaluated concerning the overall project budget and the desired treatment objectives. It is essential to balance the cost with the expected performance and longevity of the media [16].

The attached growth process involves the use of carrier media, which provide a surface for the growth of the biofilm and facilitate the treatment of wastewater. Various types of common carrier media are used in IFAS, each with unique characteristics and advantages. Table 1 compares properties of various types of carrier media used in IFAS. Kaldnes K1 is one of the most widely used carrier media in IFAS. It is made of polyethylene and has a high surface area per unit volume [96]. The media has numerous attachment points for biofilm development. Kaldnes K1 offers high durability and low clogging potential, making it suitable for various wastewater treatment applications. It provides an ideal environment for microbial growth and offers excellent treatment performance regarding organic matter and nutrient removal. Biocarrier is another popular carrier media in IFAS. It is also made of polyethylene and provides a high surface area for biofilm attachment. The media has a porous structure, which allows for efficient oxygen transfer and optimal biomass retention [96,97].

Matala Media is a polyester-based carrier media. It offers a moderate-to-high surface area per unit volume and has a porous structure. The media provides favorable conditions for the development of the biofilm and facilitates the treatment of wastewater. Matala Media exhibits good durability and low clogging potential, making it suitable for extended operational periods. Coir pith, or coconut fiber, is a fibrous carrier media. It has a moderate surface area and a fibrous biofilm attachment structure. Coir pith provides an environment conducive to microbial growth and contributes to the effective removal of organic matter in wastewater. It has low durability but offers moderate resistance to clogging. Cermedia is a ceramic-based carrier media with a high surface area per unit volume due to its porous structure. Cermedia provides an excellent substrate for biofilm growth and ensures an efficient treatment performance. Aqua Kaldness K3 is a polyethylene-based carrier media with a high surface area per unit volume. It offers attachment points for biofilm development and provides favorable conditions for microbial growth. Bioflo is a carrier media made of polypropylene that provides a high surface area for biofilm attachment and offers attachment points for microbial growth. Bioflo ensures an efficient treatment performance, durability, and low clogging potential.

The choice of carrier media in IFAS depends on factors such as surface area, attachment mechanism, durability, clogging potential, and specific treatment objectives. The carrier media discussed in this literature review offer a range of characteristics and advantages, making them suitable for different applications in wastewater treatment. Careful consideration of these factors and comprehensive pilot studies are crucial for selecting the most appropriate carrier media for the IFAS system to ensure optimal treatment performance and long-term operational stability.

### 3.6. Temperature

Temperature and seasonal variations can significantly impact the performance of IFAS. The biological processes and microbial activity are highly influenced by temperature, affecting the treatment efficiency and overall system stability [25]. As temperature increases, microbial activity and metabolic rates increase, enhancing organic matter and nutrient removal. Higher temperatures promote the growth of thermophilic bacteria, which degrade certain compounds more efficiently. However, extreme temperatures, both hot and cold, have negative impacts on microbial activity and reduce treatment performance [98,99].

Temperature influences the rates of biochemical reactions involved in wastewater treatment. The rate of biological reactions, such as nitrification and denitrification, is highly temperature dependent. Nitrification, the conversion of ammonia to nitrate, is favored at higher temperatures, while denitrification, the reduction of nitrate to nitrogen gas, is favored at lower temperatures [100,101]. Warmer temperatures result in decreased oxygen solubility, which leads to oxygen limitations. Inadequate oxygen supply hinders the performance of aerobic processes, such as organic matter oxidation and nitrification. Increased aeration and dissolved oxygen control strategies may be required to compensate for reduced oxygen transfer at higher temperatures. Temperature influences the attachment and growth of biomasses on the carrier media in the IFAS [102]. Lower temperatures reduce biofilm formation and attachment, impacting the biomass retention capacity. Operational adjustments, such as increased recirculation or hydraulic retention time, may be necessary to maintain biomass retention during colder seasons [103].

The application of IFAS is mainly in cold countries where low-temperature limits nitrogen removal. The presence of carrier media inside the bioreactor can fulfill the purpose of year-round nitrification. The addition of carrier media into the bioreactor addresses key issues limiting the efficiency of ASPs [43]. The configuration’s primary function is to complete nitrification and achieve additional treatment capacity for the wastewater treatment plant [51,104]. A detailed study on the IFAS has achieved complete nitrogen removal through nitrification/denitrification [6] and hybrid processes resulting in biological nitrogen and phosphorous removal to acceptable limits [105].

Temperature variations impact the stability and resilience of the microorganisms present in IFAS. Sudden changes in temperature, such as during seasonal transitions, disrupt the microbial community and reduce system stability. Consequently, the system may experience decreased treatment performance and longer recovery periods. Proper monitoring and control of operational parameters, including temperature, help mitigate the effects of seasonal variations and maintain stable IFAS operation. The effect of temperature and seasonal variations on IFAS performance varies depending on the system design, wastewater characteristics, and regional climate. Comprehensive monitoring, data analysis, and operational adjustments are necessary to optimize IFAS performance throughout the year and ensure consistent treatment efficiency.

### 3.7. Dissolved Oxygen

The availability of oxygen is crucial for the activity of aerobic microorganisms responsible for organic matter oxidation, nitrification, and other biological processes [57]. Sufficient dissolved oxygen is essential to support the metabolic activity of aerobic microorganisms. Adequate oxygen levels enable the efficient oxidation of organic pollutants and the conversion of ammonia to nitrate during nitrification. Higher dissolved oxygen concentrations increase microbial activity and metabolic rates, improving treatment performance [106]. Insufficient dissolved oxygen levels limit the oxidation of organic matter, resulting in decreased removal efficiency. Low DO conditions lead to incomplete degradation of organic pollutants, reducing treatment effectiveness and accumulating residual organic compounds. Maintaining optimal DO levels allows for effective organic matter removal and prevents the build-up of recalcitrant compounds [107].

Nitrification requires a sufficient supply of dissolved oxygen to support the growth and activity of nitrifying bacteria. Inadequate DO levels can inhibit nitrification, leading to decreased ammonia removal and potential ammonia toxicity issues downstream. On the other hand, denitrification, the reduction of nitrate to nitrogen gas, occurs under low dissolved oxygen conditions. Controlling DO levels in IFAS systems is crucial for balancing nitrification and denitrification processes. Insufficient oxygen can lead to excessive nitrate accumulation and hinder denitrification. Optimizing dissolved oxygen levels in different process zones allows for efficient nitrogen removal [108,109].

DO levels influence biofilm growth on the carrier media. Higher DO concentrations promote the formation of thicker and more active biofilms. Well-developed biofilms enhance the treatment efficiency by providing a large surface area for microbial attachment and allowing for better pollutant degradation [110,111]. Adequate dissolved oxygen levels support optimal biofilm growth and biomass activity. Maintaining adequate dissolved oxygen levels helps prevent the occurrence of anaerobic zones within the IFAS system. Anaerobic conditions can lead to the proliferation of anaerobic microorganisms, causing process instabilities, odor issues, and the production of undesirable by-products. Aerobic conditions are sustained by maintaining appropriate DO levels, preventing anaerobic zones, and ensuring stable system performance [112].

The plug-flow IFAS treats real sewage wastewater by applying a partial nitritation/anammox (PN/A) to remove nitrogen compounds efficiently. The influent contains a C/N ratio = 1.3, is rich in ammonium and nitrogen (NH_4_^+^–N 45.0 ± 5.2 and TN 54.7 ± 6.4), and obtained a TN removal of 82% at a nitrogen removal rate of 0.097 ± 0.019 kg N/(m^3^ d). The PN/A performance deteriorated with the ammonium concentration below 1 mg/L. On the other hand, stability of the PN/A was achieved in the long run when the concentration of residual ammonium was more than 3 mg/L [113]. The research was conducted on the nitritation-anammox in pilot-scale and full-scale IFAS to treat domestic wastewater. A polyesters sponge (cubic) was used as the carrier media, and the reactor was tested under rich ammonium wastewater. The presence of highly suspended solids seriously hinders performance. Pre-treatment was recommended to reduce the influent from suspended solids [114].

A pilot plant ASP to IFAS was converted to treat high-ammonium wastewater by filling an immobile carrier. The ammonium loading rate was progressively escalated to optimize the maximum removal and efficiency. The pilot plant showed promising results for removing COD, NH_4_^+^–N, and PO_4_^3−^–P, and overall removal efficiencies of 96.6%, 99.9%, and 98.8% were obtained, respectively [115]. The comparison of the MBBR and the fixed bed bioreactor (FBBR) has been made for denitrification and phosphorous removal. The results indicated that the FBBR works more efficiently than the MBBR to remove all nutrients. The MBBR removed total chemical oxygen demand (tCOD) 19.8%, filtered COD 35.5%, BOD_5_ 27.6%, acetate 62.2%, PO_4_^3−^–P 78.5%, and NO_3_^−^–N 54.2%, while the FBBR removed tCOD 49.7%, filtered COD 54.0%, BOD_5_ 63.2%, acetate 99.6%, PO_4_^3−^–P 98.6% and NO_3_^−^–N 575.9%, respectively [116].

The IFAS reactor has been implemented for the SAND for nitrogen removal from synthetic wastewater rich in ammonium. The anammox is energy-efficient, but COD in the influent stream hinders nitrogen removal efficiency through deammonification. The SNAD-IFAS achieved 72 ± 2% TN removal efficiency with a TN effluent concentration of 14.9 mg/L and an average COD removal efficiency of 88%. The system depicted these results at COD/N ratio = 1.2 ± 0.2. The activity test and illumine sequencing analysis indicated the presence of denitrifying and anammox bacteria in the biofilm conducting nitrogen removal activities [117].

The optimal dissolved oxygen levels can vary depending on the specific wastewater characteristics, process design, and operational conditions of the IFAS system. Regular monitoring of DO levels and adjustments to aeration rates or oxygen transfer mechanisms are necessary to maintain optimal dissolved oxygen concentrations for efficient treatment performance and to avoid oxygen limitation and excessive energy consumption.

## 4. IFAS Combined with Membrane Technology

IFAS has been effectively combined with membrane technology to enhance treatment efficiency and overall performance. The combination, IFAS-MBR, offers several advantages regarding process stability, effluent quality, and system compactness. Adding IFAS to an MBR provides a larger biomass population and surface area for the growth of nitrifying and denitrifying bacteria [118]. It promotes enhanced nutrient removal, specifically nitrogen, and phosphorus. The IFAS media acts as a substrate for the attachment and growth of a biofilm, facilitating the conversion of ammonia to nitrate and the subsequent denitrification process. The combination is particularly beneficial for meeting stringent effluent nutrient discharge limits [55].

The biofilm on the IFAS media provides a stable and resilient microbial population that withstands fluctuations in influent wastewater characteristics. The addition of the membrane further enhances process stability by physically separating the biomass from the treated effluent. It prevents the washout of the biomass and ensures consistent treatment performance even during hydraulic and organic load variations. The membrane is a physical barrier, effectively retaining biomass and suspended solids within the bioreactor. It leads to a higher sludge retention time (SRT) and improved solids retention, enhancing biological treatment efficiency. Combining IFAS and membrane technology enables a higher biomass concentration, reducing the system’s footprint and providing a compact and efficient treatment solution [48,63].

Mannina et al. [119] ran experiments for the IFAS-MBR hybrid system to remove carbon, nitrogen, and phosphorous. The system provided excellent total COD removal efficiencies, and an average removal efficiency of 98% was obtained throughout the experiments. The results are the same as those of the MBR and hybrid MBR configurations [120]. Both of the systems contain the same filling ratio of carrier media and HRT values, achieving more than 98% of the removal of organics [121]. The organics removal for the IFAS is slightly higher when compared with hybrid MBBR-MBR, utilizing different carrier media for both of the reactors. The reason for this is the difference in the operating conditions as the latter was operated at lower HRT and low C/N ratio of 3–5 and a higher membrane pore size (0.1 µm). The results obtained by different researchers for the IFAS/IFAS-MBR systems confirm the robustness of both systems for organic matter removal [122,123,124].

The MBBR and IFAS achieved >90% COD removal efficiency with an effluent concentration below 50 mg/L. They treated domestic wastewater to remove COD and concluded that low values of DO are not the limiting factor [125]. The low-temperature values do not affect the IFAS performance, even when lower than 9 °C and lower pollutant concentrations (snow melting) [78]. Removing trace organics in three configurations: MBR, IFAS-MBR, and moving bed membrane bioreactor (MBMBR) was researched to investigate the overall efficiency. The IFAS-MBR was operated at a 50% filling ratio with a 13 h HRT and 20 d SRT. The results were compared with MBBR-MBR, which has lower removal efficiency due to a lower MLSS concentration and a lower lowest value of HRT (6 h) [126].

The biofilm formation and sludge cake for SFD-MBR coupled with IFAS were studied to improve denitrification. A PE carrier with a specific surface area of 489 m^2^/m^3^ and a filling ratio of 19% was used at a DO level of 2–4 mg DO/L, 8 h HRT and 30 d SRT. The SFD-MBR and IFAS coupled with SFD-MBR showed similar performance in productivity relationships, organics oxidation, and ammonium compounds. The IFAS-SFD-MBR produces less sludge and improves denitrification but requires more frequent mesh cleaning because the carrier media negatively affects the tendency of mesh clogging, subsequently leading to a three-time increase in cleaning frequency. The attached growth on carrier media resulted in less sludge production, and the presence of anoxic conditions inside the carrier media improves the denitrification reducing the effluent nitrate concentration [118].

Two MBMBRs were operated to investigate the effect of carrier media on membrane fouling mitigation. It was observed that the membrane filtration operating time for the MBMBR (without the scouring of carrier) extended by 1.5 times compared to the MBR. With the carrier media’s scouring, the time was extended by eight times. A comparison of the MBR and MBMBR depicted that membrane fouling decreased by 58.8%. The cake layer fouling was decreased to 40.5% for MBMBR_sc_, thanks to the scouring by carrier media [127].

The pilot-scale hybrid IFAS-MBR has been studied for bacterial community structure and organics removal from real municipal wastewater. It reached a maximum removal efficiency for COD at 98%, nitrification at 98%, and an average TP removal efficiency of 40.4%. The IFAS-MBR pilot plant depicted excellent COD removal efficiency, despite variations of the influent, with average values of >98%. The 98% nitrification result indicates biofilm activity. Membrane fouling was examined due to superficial cake deposition. Biofilm detachment increases the pore-blocking resistance due to an increased EPS fraction [128].

Anaerobic moving bed biofilm reactor (AMBBR) and IFAS-SBR were focused on creating a novel A-B configuration, with ‘A’ being AMBBR for COD reduction-producing biogas and IFAS-SBR as stage ‘B’ for nitrogen removal. This system removed about 85% of COD with a total energy production rate of 0.28 kWh/m^3^ while achieving 85% nitrogen removal. Sludge production decreased by about 75% compared to conventional ASPs [129]. Consequently, this combination is ideal for attaining energy-neutral or energy-positive operation of the wastewater treatment plant. The mainstream wastewater treatment in IFAS by the PN/A has been investigated and compared with the performance of the MBBR reactor [27]. Conversion of the MBBR to IFAS increases nitrogen removal—a full-scale IFAS plant to demonstrate nitrogen removal assessment through nitrification. The plant treats 76,000 m^3^/day of wastewater through a modified Ludzack-Ettinger (MLE) configuration [16].

The IFAS-MBR offers system design flexibility, allowing various configurations to suit wastewater treatment needs. The IFAS media and membrane modules are integrated into the bioreactor, providing options for different types of carrier media and membrane configurations. This flexibility enables customization based on site-specific requirements, including influent characteristics, treatment goals, and available space. The IFAS-MBR offers several advantages, including enhanced nutrient removal, improved process stability, superior effluent quality, reduced sludge production, and flexible system design. This combination is particularly beneficial for applications that require advanced wastewater treatment and strict effluent discharge standards.

## 5. Comparison of ASP, IFAS, and IFAS-MBR

ASPs, IFAS, and IFAS-MBRs have widely used wastewater treatment processes with performance aspects [130]. An ASP is a suspended growth process that relies on the biological activity of microorganisms in a mixed liquor suspended in wastewater. It offers moderate-to-high organic matter removal and medium nutrient removal efficiency. However, it requires a large footprint and space due to the need for large settling tanks, and the sludge production is moderate. The ASP operates with basic process control and automation, making it a reliable but less advanced option. Table 2 compares different aspects of ASPs, IFAS, and IFAS-MBRs for wastewater treatment.

The IFAS introduces attached biofilm carriers into the suspended growth process, enhancing the treatment efficiency. It provides moderate-to-high organic matter removal and high nutrient removal efficiency. Adding biofilm carriers improves solids retention and reduces sludge production compared to an ASP. The IFAS has a reasonable footprint and space requirement due to the biofilm carriers. It also offers lower energy consumption and high capital and operating costs than an ASP [126].

The IFAS-MBR combines the advantages of IFAS with membrane bioreactor technology. It achieves high organic matter removal and high nutrient removal efficiency. The membrane filtration in the IFAS-MBR ensures excellent solids retention and produces low sludge volumes. It provides advanced process control and automation, achieving high stability and reliability. However, the IFAS-MBR requires more increased capital investment and has moderate operating costs. The energy consumption is low, and the effluent quality is high [28,59,85].

The selection of the most suitable process depends on effluent quality requirements, available land, regulatory constraints, and lifecycle costs. The ASP is a well-established process with moderate performance, while IFAS offers improved treatment efficiency and reduced sludge production. The IFAS-MBR provides advanced treatment and high-quality effluent but requires a higher capital investment. Careful consideration of these performance aspects is essential in choosing the optimal process for a specific wastewater treatment application [55,131].

## 6. Future Prospective and Conclusions

The adoption of IFAS is expected to increase due to its effectiveness in treating wastewater and its potential to reduce the environmental impact of wastewater treatment. IFAS can be used for decentralized wastewater treatment systems, providing a cost-effective and sustainable solution for rural and remote areas where centralized treatment systems are not feasible. Researchers are working to improve the efficiency of IFAS technology by optimizing the design and operation of the fixed-film system and enhancing the biofilm properties. IFAS technology can provide a cost-effective and sustainable solution for wastewater treatment in developing countries where access to wastewater treatment is limited. IFAS technology can be used for treating wastewater in various industries, including food and beverage, pulp and paper, and pharmaceuticals, to name a few.

IFAS technology can be integrated with other treatment processes, such as membrane filtration, to create a highly efficient and effective wastewater treatment plant. The combination of IFAS and MBR technology provides a high level of treatment performance and can produce a high-quality effluent suitable for reuse or discharge to sensitive environments. The IFAS can help reduce the fouling of the MBR membranes and increase the system’s treatment capacity. The integration of IFAS with anaerobic digestion can help to enhance the production of biogas and increase the removal of organic matter and nutrients. The IFAS can provide a pre-treatment step that helps improve the wastewater quality before it enters the anaerobic digester. IFAS can be integrated with denitrification filters to enhance nitrogen removal from wastewater. The IFAS provides a source of carbon that can be used by denitrifying bacteria to convert nitrate to nitrogen gas, which is then released into the atmosphere. Integrating IFAS with chemical precipitation can help remove phosphorus from wastewater. The IFAS provides a source of organic matter that can be used by phosphorus-removing bacteria to facilitate the removal of phosphorus from wastewater.

IFAS has been widely used in water reuse applications because it provides a high quality effluent suitable for various non-potable reuse applications, such as irrigation, industrial uses, and environmental enhancement. The use of IFAS in water reuse applications has several benefits, including providing a sustainable and reliable water source, reducing the demand for freshwater resources, and protecting the environment by reducing the discharge of treated wastewater to surface waters. Additionally, the IFAS in water reuse applications can save costs compared to traditional treatment, such as reverse osmosis. Overall, integrating IFAS technology with other treatment processes can help enhance the treatment performance, increase the treatment capacity, and improve the overall efficiency of the wastewater treatment system. The selection of the appropriate treatment processes to integrate with IFAS depends on the specific wastewater characteristics and treatment goals.

Various operating parameters influence the performance of IFAS. Optimal control and management of these parameters are crucial to achieve efficient pollutant removal and maintain system stability. A longer HRT allows for more contact time between microorganisms and pollutants, enhancing removal efficiency. Typical values of HRT are 4–8 h for organic matter removal and 8–12 h for nutrient removal. An appropriate SRT ensures sufficient biomass concentration for efficient pollutant degradation. SRT values of 5–20 d are employed, depending on the specific treatment goals and wastewater characteristics. Adequate DO promotes aerobic microbial activity and improves pollutant degradation. DO levels of 2–3 mg/L for organic matter removal, and 1–2 mg/L for nitrification are typically used for wastewater treatment. Higher temperatures generally enhance microbial activity and biochemical reactions, improving treatment efficiency. A temperature of 20–35 °C is mostly used for optimal performance, but it varies based on specific microbial populations and process requirements.

This review of the effect of operating parameters on IFAS systems for wastewater treatment provides valuable insights into optimizing system performance and achieving efficient pollutant removal. The findings underscore the importance of considering various operating parameters, such as hydraulic retention time, solids retention time, oxygen supply, temperature, and aeration, to enhance treatment efficiency and maintain system stability. By carefully controlling these parameters, the IFAS can effectively remove organic matter and nutrients, improving effluent quality. Furthermore, optimizing operating parameters helps reduce energy consumption, minimize sludge production, and enhance overall treatment process sustainability. However, further research is needed to better understand the complex interactions between operating parameters and their impact on IFAS performance. It includes investigating the influence of different wastewater characteristics, evaluating the long-term effects of parameter variations, and exploring the potential for advanced process control and resource recovery. In conclusion, by considering and optimizing operating parameters, IFAS can be designed and operated to achieve high-performance wastewater treatment, providing a sustainable and efficient solution for addressing water pollution challenges.

IFAS has the potential to be used in a variety of applications, including municipal and industrial wastewater treatment, decentralized treatment systems, and in developing countries where access to wastewater treatment is limited. IFAS provides a cost-effective and sustainable solution for these applications. In conclusion, IFAS is an innovative and effective method for treating wastewater. Its potential to reduce the environmental impact of wastewater treatment and be integrated with other treatment processes makes it a promising technology for the future of wastewater treatment.

## Figures and Tables

**Table 1:** Comparison of properties of various types of carrier media used in IFAS.

Carrier Media	Surface Area (m^2^/m^3^)	Durability	Clogging Potential	Cost
Kaldnes K1	500–700	High	Low	Moderate
Biocarrier MBBR	400–600	High	Low	Moderate
AnoxKaldnes MBBR	500–800	High	Low	Moderate
Matala Media	150–200	Moderate	Low	Low
Coir Pith	100–150	Low	Moderate	Low
Cermedia	800–1000	High	Low	High
Aqua Kaldness K3	800–1000	High	Low	Moderate
Bioflo	600–800	High	Low	Moderate

**Table 2:** Comparison of ASP, IFAS, and IFAS-MBR for wastewater treatment.

Aspect	Criteria	IFAS-MBR	IFAS	ASP
Treatment Efficiency	Organic matter removal (%)	95–99%	80–90%	70–90%
	Nutrient removal (%)	90–95% (N), 85–90% (P)	70–85% (N), 50–70% (P)	30–60% (N), 20–50% (P)
	Pathogen removal efficiency	High	Moderate	Moderate
	Solids removal efficiency	99%	95–98%	90–95%
Membrane Technology	Membrane type	Low-pressure	N/A	N/A
	Membrane fouling control	Effective	N/A	N/A
	Membrane replacement interval	5–10 years	N/A	N/A
Operational Control	Process control and automation	Advanced	Moderate	Basic
	Operator intervention	Minimal	Moderate	Moderate
	Process stability	High	Moderate	Moderate
Energy Consumption	Average energy consumption	0.5–1.0 kWh/m^3^	0.8–1.5 kWh/m^3^	1.0–2.5 kWh/m^3^
	Energy recovery efficiency	30–50%	10–20%	5–10%
Sludge Production	Excess sludge production	Low	Moderate	High
	Sludge handling and disposal	Low	Moderate	Moderate
Capital and Operating Cost	Initial capital cost	High	Moderate	Moderate
	Operational cost	Moderate	Low	Moderate
	Maintenance requirement	Moderate	Low	Moderate
	Footprint requirement	Moderate	Moderate	Large
	Scalability	High	High	Moderate
Environmental Impact	Odor generation	Low	Moderate	Moderate
	Greenhouse gas emissions	Low	Moderate	Moderate
	Chemical usage	Low	Moderate	Moderate
	Noise pollution	Low	Moderate	Moderate
	Ecological footprint	Low	Moderate	Moderate

## Data Availability

Not applicable.
